# Fascin 2b Is a Component of Stereocilia that Lengthens Actin-Based Protrusions

**DOI:** 10.1371/journal.pone.0014807

**Published:** 2011-04-26

**Authors:** Shih-Wei Chou, Philsang Hwang, Gustavo Gomez, Carol A. Fernando, Megan C. West, Lana M. Pollock, Jennifer Lin-Jones, Beth Burnside, Brian M. McDermott

**Affiliations:** 1 Department of Otolaryngology–Head and Neck Surgery, Case Western Reserve University School of Medicine, Cleveland, Ohio, United States of America; 2 Department of Biology, Case Western Reserve University, Cleveland, Ohio, United States of America; 3 Department of Genetics, Case Western Reserve University School of Medicine, Cleveland, Ohio, United States of America; 4 Department of Neurosciences, Case Western Reserve University School of Medicine, Cleveland, Ohio, United States of America; 5 Department of Molecular and Cell Biology, University of California, Berkeley, California, United States of America; University of Birmingham, United Kingdom

## Abstract

Stereocilia are actin-filled protrusions that permit mechanotransduction in the internal ear. To identify proteins that organize the cytoskeleton of stereocilia, we scrutinized the hair-cell transcriptome of zebrafish. One promising candidate encodes fascin 2b, a filamentous actin-bundling protein found in retinal photoreceptors. Immunolabeling of zebrafish hair cells and the use of transgenic zebrafish that expressed fascin 2b fused to green fluorescent protein demonstrated that fascin 2b localized to stereocilia specifically. When filamentous actin and recombinant fusion protein containing fascin 2b were combined *in vitro* to determine their dissociation constant, a *K*_d_≈0.37 µM was observed. Electron microscopy showed that fascin 2b-actin filament complexes formed parallel actin bundles *in vitro*. We demonstrated that expression of fascin 2b or espin, another actin-bundling protein, in COS-7 cells induced the formation of long filopodia. Coexpression showed synergism between these proteins through the formation of extra-long protrusions. Using phosphomutant fascin 2b proteins, which mimicked either a phosphorylated or a nonphosphorylated state, in COS-7 cells and in transgenic hair cells, we showed that both formation of long filopodia and localization of fascin 2b to stereocilia were dependent on serine 38. Overexpression of wild-type fascin 2b in hair cells was correlated with increased stereociliary length relative to controls. These findings indicate that fascin 2b plays a key role in shaping stereocilia.

## Introduction

The senses of hearing and equilibrium in vertebrates depend on the mechanosensitive hair bundle, which consists of a precise arrangement of actin-based stereocilia that extend from the hair cell's apical surface [1]–[5]. A systematic increase in stereociliary length results in a beveled hair bundle. Each cylindrical stereocilium is stiff, but its uniform girth tapers towards the base to allow for flexion. These attributes, combined with extracellular linkages that tether stereocilia together, allow the bundle to move as a single unit [6].

Actin cross-linking proteins are necessary for the proper rigidity, length, and thickness of stereocilia [7]. The actin filaments in stereocilia are highly ordered by cross-linking proteins [8]–[10]. In the core of each stereocilium, a dense actin-based rootlet extends through the tapered region into the cuticular plate to anchor the protrusion [11]. Two actin-bundling proteins are known to be present in stereocilia: fimbrin [12], [13] and espin [14]–[16]. The latter is required for hearing in humans and in mice. Espin allows strands of filamentous actin to be bundled into parallel actin bundles in biochemical assays and in cultured cells. In addition, espin has been shown to participate in the elongation of stereocilia [17]. Finally, the actin core of each stereocilium is thought to undergo continuous treadmilling. During this process, actin polymerization and espin-mediated cross-linking take place at the distal (or barbed) ends, and depolymerization occurs at the pointed ends of the actin filaments; this results in rearward movement of the filaments [7], [18], [19].

Fascins constitute a family of monomeric proteins that organize actin filaments into well-ordered, tightly packed, parallel bundles that participate in the cytoskeletal organization of cell surface protrusions and somatic bundles [20]. Generally, vertebrates have three fascin genes, numbered 1, 2, or 3, which encode proteins with highly similar amino acid sequences [20]. Zebrafish have two fascin 2 paralogs, *fascin 2a* and *fascin 2b* [21]. Fascin 2b is expressed in photoreceptors, in which it has been implicated in the bundling of actin [21]. Each fascin protein has four fascin domains and a highly conserved region between residues 11 and 50, which contains a site for protein kinase C (PKC) phosphorylation at serine 39 in fascin 1 [20] and a putative PKC phosphorylation site at serine 38 in fascin 2b [21]. Mutational analyses of fascins have shown that phosphorylation of serine 39 or 38 regulates actin binding by fascin 1 or 2b [21]–[23], respectively, and fascin 1 localization to cell surface protrusions [24], [25]. Fascin 1 is thought to have two actin-binding sites: one that maps near the C-terminus [22], [23] and another towards the N-terminus in a region with high sequence similarity to an actin-binding site of myristoylated alanine-rich C-kinase substrate (MARCKS) [20]. This MARCKS-like region, which putatively interacts with actin, overlaps with the PKC phosphorylation site. Phosphorylation of serine 39 inhibits both actin-binding and actin-bundling activities of fascin 1. Conversely, removal of the phosphorylation site at serine 39, by replacing the serine with an alanine, allows actin binding *in vitro* [22], [23].

Based on our search of the hair-cell transcriptome, we here characterize fascin 2b as a candidate protein that organizes stereocilia. Using RNA *in situ* hybridization and immunolabeling, we show that fascin 2b is a component of stereocilia in zebrafish hair cells. By the expression of wild-type or phosphomutant fascin 2b proteins, we establish that fascin 2b can induce the formation of long filopodia, and these protrusions are dependent on serine 38. Furthermore, our studies using transgenic zebrafish indicate a reliance on serine 38 for localization of fascin 2b to stereocilia. Moreover, we demonstrate that overexpression of fascin 2b in hair cells results in longer stereocilia when compared to control cells. We conclude that fascin 2b is important for the morphology of stereocilia, and this protein's function in cells is governed by the phosphorylation state of serine 38.

## Results and Discussion

### *Fascin 2b* mRNA is the predominant fascin 2 mRNA expressed in zebrafish hair cells

**Figure 1:**
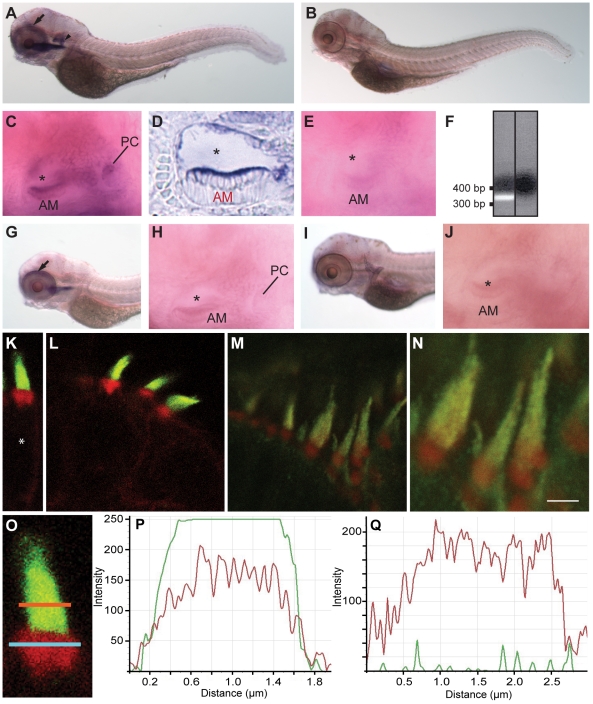
Localization patterns of fascin 2 mRNAs and proteins in zebrafish. RNA *in situ* hybridizations (**A–E**, **G–J**) were performed on larvae at 4 dpf. *Fascin 2b* mRNA is detected in the otocysts (**A**, arrowhead; **C**) and the eye (**A**, arrow). *Fascin 2a* mRNA is expressed in the eye (**G**, arrow), but not the otocyst (**H**). *Fascin 2b* sense RNA (**B**,**E**) and *fascin 2a* sense RNA (**I**,**J**), each labeled, were used as controls. A cryosectioned ear labeled with *fascin 2b* antisense probe is displayed (**D**). AM, PC, and asterisk indicate the anterior macula, the posterior crista, and the lumen of the otocyst, respectively (**C**,**D**,**E**,**H**,**J**). RT-PCR analysis (**F**) shows expression of *fascin 2b* in zebrafish hair cells. Agarose gel confirms the expected product size of the *fascin 2b* amplicon, left lane (+ hair-cell cDNA); no product is observed without cDNA template, right lane (no template control). Labeling using fascin 2 antiserum reveals strong fluorescent signals (green) in hair bundles of an anterior macula (**K**,**O**) and a posterior crista (**L**) from larvae at 4 dpf and of an adult lagena (**M**,**N**). In red, fluorophore-coupled phalloidin labels the filamentous actin of stereocilia and cuticular plates (**K–O**). Higher magnification of **M** is displayed (**N**). Scale bar is 2 µm. Soma is indicated by asterisk (**K**). An enlarged view of a hair bundle from an anterior macula (**O**) is shown with regions of interest (ROI) selected for the stereocilia (orange line) and the cuticular plate (blue line). Fluorescence intensity profiles of stereocilia (**P**), using the orange-line ROI from **O**, show that the fascin 2b- (green) and phalloidin-associated (red) signals are overlapping. Intensity profiles of the cuticular plate (**Q**), using the blue-line ROI from **O**, demonstrate no significant labeling with fascin 2 antiserum (green). Intensity scales are linear, but the units are arbitrary (**P**,**Q**). *X*-axes (**P**,**Q**) represent the lengths of the respective orange and blue lines (**O**).

**Figure 2:**
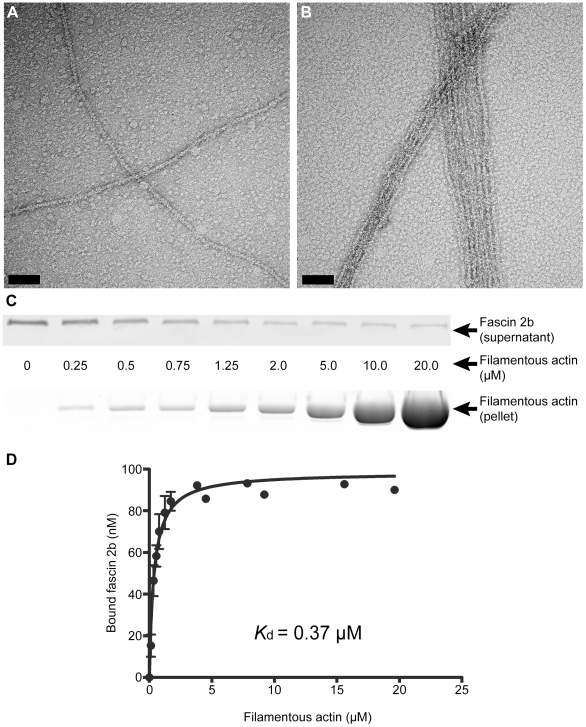
Physical characterization of the interaction between fascin 2b and filamentous actin. Negative staining and electron microscopy reveal actin bundling by recombinant fascin 2b fusion protein. Micrographs display filamentous actin in the absence of MBP-fascin 2b (**A**) and in the presence of MBP-fascin 2b (**B**); bundled filaments are observed in **B**. Scale bars are 100 nm. An immunoblot (**C**, top), from a cosedimentation experiment, shows the progressive depletion of MBP-fascin 2b from supernatants incubated with increasing concentrations of actin; molar concentration (**C**, middle) of actin for each lane is displayed. Image of a gel (**C**, bottom), after SDS-PAGE and exposure to SYPRO Ruby protein gel stain, shows the actin found in pellets from a cosedimentation experiment. Plotted are the means of specific equilibrium-binding measurements of MBP-fascin 2b to filamentous actin (**D**). Each point is a mean ± SEM (number of experiments, N = 4) or an average of two experiments (no error bars).

To identify proteins important for the development, maintenance, and function of stereocilia, we searched the zebrafish hair-cell transcriptome [26] for expressed genes that may encode proteins that bundle filamentous actin. One candidate identified was the product of the *fascin 2b* gene, which encodes a protein that has a known role in photoreceptors as an actin cross-linker [21]. We hypothesized a function for fascin 2b in the organization of actin in the hair bundle. To confirm the presence of *fascin 2b* mRNA in the zebrafish ear, we performed whole-mount *in situ* hybridization studies on larvae at 4 days postfertilization (dpf) (**Figure 1A**) and 2 dpf (data not shown); higher magnification revealed expression specific to the anterior maculae and the posterior cristae (**Figure 1C**) of larvae. Cryosections of these whole-mount preparations confirmed expression in anterior maculae (**Figure 1D**). This expression pattern was similar in appearance to those observed for other hair cell-specific mRNAs [27], [28]. In addition, we performed whole-mount *in situ* hybridization experiments that probed for *fascin 2a* mRNA; no expression was detected in the ears (**Figure 1G,H**). However, both *fascin 2a* mRNA and *fascin 2b* mRNA were expressed in the eyes (**Figure 1A,G**). The expression patterns of *fascin 2a* at 2 and 4 dpf were similar; in addition, the tissues that expressed *fascin 2b* were essentially the same at both time points (data not shown). We performed reverse transcription-polymerase chain reactions (RT-PCR) using RNA collected exclusively from adult zebrafish hair cells [26] and demonstrated the presence of *fascin 2b* mRNA (**Figure 1F**). We also detected *fascin 2a* mRNA in hair cells of the adult ear by RT-PCR (**Figure S1**). However, because *fascin 2a* mRNA was not detectable by *in situ* hybridization in the ears of larvae at 2 or 4 dpf, we conclude that *fascin 2b* mRNA is the abundant fascin 2 transcript present in hair cells at these developmental stages. As negative controls, samples were included to determine whether mRNAs that are expressed in the liver could be identified in the hair-cell RNA preparation [26]. The liver mRNAs encoded by *apolipoprotein A-I* and *apolipoprotein Eb* are known to be absent from hair cells [26], and these reactions showed no products (**Figure S1**). These liver mRNAs were clearly detectable by RT-PCR when cDNA from whole larvae was used as template (data not shown). To further substantiate our finding that *fascin 2b* mRNA is the prevalent fascin 2 transcript expressed in zebrafish hair cells, we performed absolute quantitative real-time polymerase chain reaction experiments [29]. These studies indicated that in adult zebrafish hair cells *fascin 2b* mRNA was 44 times more abundant than *fascin 2a* mRNA (**Figure S2**).

### Localization of fascin 2b to the actin bundles of stereocilia

Because proteins of the fascin family localize to actin-based protrusions with great specificity [30]–[32], we hypothesized that fascin 2b may localize to stereocilia and assist in assembly and/or stabilization of the hair bundle. Whole zebrafish larvae at 4 dpf were labeled with an antiserum that recognizes both fascin 2a and fascin 2b [21] and were decorated with fluorophore-coupled phalloidin for imaging the densely packed actin of stereocilia. We demonstrated that the antiserum localized to larval stereocilia with high specificity in anterior maculae (**Figure 1K**), cristae (**Figure 1L**), and posterior maculae (data not shown); no other regions of the hair cell were significantly labeled with the antiserum. Because only *fascin 2b* mRNA was observed in hair cells by *in situ* hybridization studies, the antiserum likely detected fascin 2b and not fascin 2a. In addition, we determined that fascin 2 antiserum labeled stereocilia of adult zebrafish lagenae, which are otolithic end organs (**Figure 1M,N**). Fluorescence intensity profiles showed that fascin 2b immunolabeling overlapped with fluorophore-coupled phalloidin in stereocilia (**Figure 1O,P**). This result contrasted with the cuticular plate fluorescence intensity profiles, in which no significant fascin 2b labeling was observed (**Figure 1O,Q**). Fascin 2b was not detected in the lateral-line system (data not shown). This absence may indicate a lack of fascin 2b expression in hair cells of the lateral line; however, it could also be an artifact of direct exposure of these surface cells to the harsh fixative, which may have destroyed the epitope.

### Fascin 2b and filamentous actin form highly ordered bundles when combined *in vitro*

A salient property of actin-bundling proteins that localize to stereocilia is that they organize strands of filamentous actin into parallel bundles [13], [33], [34]. Recombinant fascin 2b protein can cause actin bundling *in vitro* [21], but it is not known if this bundling forms loose, orthogonal networks, like those that can be formed by filamin [35], or regular, closely packed, parallel actin bundles as in stereocilia. To examine actin bundling by fascin 2b, this protein was expressed with an N-terminal maltose-binding protein (MBP) tag (MBP-fascin 2b) and then purified from bacterial lysates [21]. MBP alone does not bind or bundle actin [21]. We combined actin that was isolated from rabbit skeletal muscle with recombinant fascin 2b in filamentous buffer, negatively stained the sample, and then viewed it using transmission electron microscopy (**Figure 2**). The ratio of recombinant fascin 2b fusion protein to monomeric actin was 2.1 to 1. The electron micrographs showed the formation of regular, closely packed actin bundles with filaments bearing a centerline arrangement, characteristic of parallel actin bundles (**Figure 2A,B**). This indicates that fascin 2b causes the formation of highly ordered bundles of filamentous actin rather than loose networks.

### The binding affinity of fascin 2b and filamentous actin is on par with that of espin and filamentous actin

To further understand the interaction of fascin 2b with filamentous actin and to compare it with the espin-actin interaction [33]–[37], we determined the filamentous actin-MBP-fascin 2b dissociation constant (*K*_d_) using the established supernatant-depletion methodology [38], [39]. More specifically, through western blot analyses and the quantitation of protein in denaturing polyacrylamide gels, we calculated the amounts of actin-bound fascin 2b depleted from supernatants after ultracentrifugation (**Figure 2C,D**). The *K*_d_ was determined to be ≈0.37 µM(**Figure 2C,D**). This binding affinity is similar to that of the espin-filamentous actin interaction, which has a *K*_d_≈0.22 µM [33]. This *in vitro* interaction study, along with the electron micrographs (**Figure 2A,B**), indicates that fascin 2b and espin may interact similarly with filamentous actin in stereocilia; however, their modes of regulation differ [16], [17], [21], and this may suggest that these proteins have distinct roles in these protrusions.

### Long filopodia are generated when fascin 2b is expressed in COS-7 cells, and the effect is augmented by coexpression with espin

**Figure 3:**
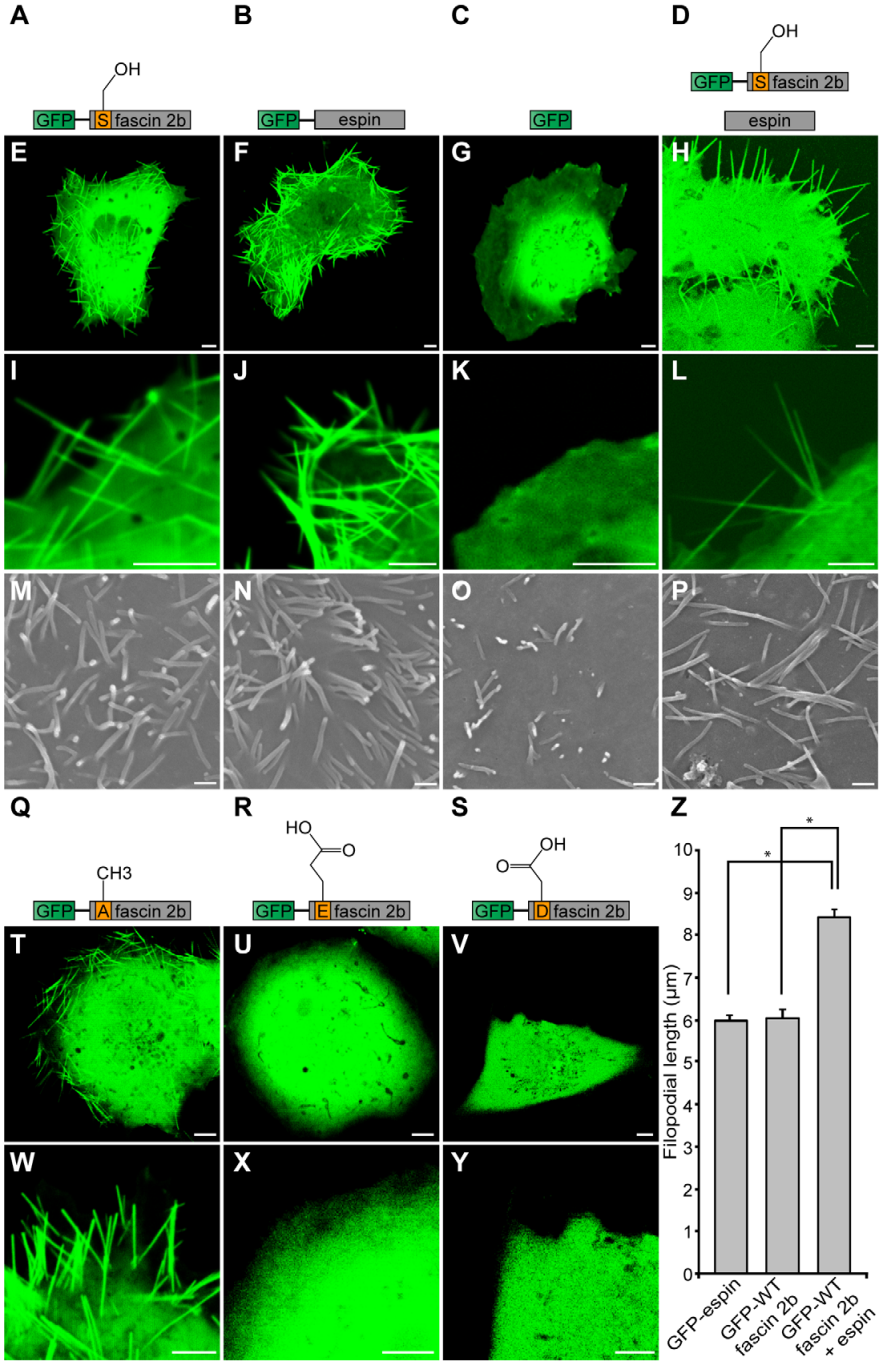
Roles of wild-type or phosphomutant fascin 2b proteins in the formation of long filopodia. Schematics of proteins expressed in COS-7 cells and corresponding confocal micrographs are displayed: GFP-WT fascin 2b (**A**,**E**,**I**), GFP-espin (**B**,**F**,**J**), GFP (**C**,**G**,**K**), GFP-WT fascin 2b and espin (**D**,**H**,**L**), GFP-S38A fascin 2b (**Q**,**T**,**W**), GFP-S38E fascin 2b (**R**,**U**,**X**), or GFP-S38D fascin 2b (**S**,**V**,**Y**). Confocal images are of representative live cells. Filopodia are observable in cells expressing GFP-WT fascin 2b (**E**,**I**), GFP-espin (**F**,**J**), or GFP-S38A fascin 2b (**T**,**W**). None are apparent in cells in which only GFP was introduced, as exhibited by this representative cell (**G**,**K**). Cells expressing GFP-S38E fascin 2b (**U**,**X**) or GFP-S38D fascin 2b (**V**,**Y**) have no filopodia visible by confocal microscopy. Cells that express both GFP-WT fascin 2b and espin have longer cytoplasmic protrusions (**H**,**L**,**Z**) than cells that express GFP-WT fascin 2b or GFP-espin separately (**E**,**F**). Scanning electron micrographs of cells expressing GFP-WT fascin 2b (**M**), GFP-espin (**N**), or GFP-WT fascin 2b and espin (**P**) show results similar to those of cells observed using confocal microscopy, except that diminutive filopodia are seen on the surfaces of cells that express the GFP control, as shown in image (**O**). The mean lengths ± SEM of the cellular protrusions are shown for cells expressing GFP-espin (N_filopodia_ = 400), GFP-WT fascin 2b (N_filopodia_ = 130), or GFP-WT fascin 2b together with espin (N_filopodia_ = 330), and they indicate synergism between fascin 2b and espin (**Z**). Asterisk indicates *P*<0.0001 for Student's *t* test. Scale bars of confocal images and electron micrographs are 5 µm and 1 µm, respectively.

Some actin-associated proteins that participate in stereociliary development and maintenance have the capacity to induce the formation of long filopodia or long microvilli in cultured cells [17], [40], [41]. To test whether fascin 2b acts similarly, in COS-7 cells, we expressed wild-type fascin 2b fused to green fluorescent protein (GFP-WT fascin 2b) or fascin 2b phosphomutant fusion proteins that simulate the different states of phosphorylation at serine 38. The COS-7 cell line has been used for *in vitro* expression to reveal the fundamental attributes of proteins, including those proteins that are required for hearing [17], [42]. In COS-7 cells in which GFP alone was introduced (number of cells, N = 102), fluorescent filopodia were not observed under our culturing conditions (**Figure 3C,G,K**). We expressed GFP-WT fascin 2b fusion protein in COS-7 cells and observed the formation of long filopodia (mean length ± standard error of the mean (SEM)  = 6.04±0.21 µm; number of filopodia, N_filopodia_ = 130; number of cells for which filopodial lengths were measured, N_cells_  = 26) (**Figure 3A,E,I**) with even distribution of GFP along the length of each protrusion. 71% of the transfected cells (N = 236) displayed long filopodia, but the remainder did not show this type of protrusion. For comparison, we expressed another actin-bundling protein, espin fused to GFP (GFP-espin) [34], in COS-7 cells and observed filopodia (5.97±0.11 µm; N_filopodia_ = 400; N_cells_  = 80) (**Figure 3B,F,J**) with lengths similar to those of cells that expressed GFP-WT fascin 2b (**Figure 3Z**). 100% of the espin-expressing cells (N = 300) formed long filopodia. To determine how the presence of both proteins influences filopodia, we coexpressed GFP-WT fascin 2b and espin in COS-7 cells to simulate conditions in stereocilia and found the mean filopodial length to be longer (8.43±0.17 µm; N_filopodia_  = 330; N_cells_ = 66) (**Figure 3D,H,L**) than the mean filopodial lengths of cells that expressed either protein alone. The mean filopodial length was approximately 2 µm greater than those of cells that expressed either GFP-WT fascin 2b (*P*<0.0001, Student's *t* test) or GFP-espin (*P*<0.0001) (**Figure 3Z**), indicating synergism between these actin-bundling proteins. 100% of cells (N = 300) that coexpressed these proteins exhibited long filopodia. To confirm that these protrusions were filopodia and therefore composed of actin, we labeled COS-7 cells that expressed GFP-espin, GFP-WT fascin 2b, or both mCherry-espin and GFP-WT fascin 2b with fluorophore-coupled phalloidin. In all cases, the protrusions were shown to be filled with actin by homogeneous phalloidin labeling (**Figure S3**). These studies demonstrate that long filopodia are generated when fascin 2b is expressed in cultured cells, and this effect is enhanced by coexpression with espin.

Observations of the surfaces of transfected cells (**Figure 3M,N,P**) using scanning electron microscopy (SEM) were consistent with those generated by high power confocal microscopy, with the exception of cells that expressed GFP alone. In this case, only SEM revealed minute filopodia (**Figure 3O**). To determine the density of filopodia on the surfaces of transfected cells, we counted all of the protrusions in the SEM micrographs and divided these values by the total visible surface area for each group of cells. The cells with the highest mean densities of filopodia expressed GFP-WT fascin 2b (mean density ± SEM = 1.19±0.25 per µm^2^; number of cells, N = 9) or GFP-espin (1.03±0.24 per µm^2^, N = 10); however, cells that expressed both GFP-WT fascin 2b and espin had an intermediate mean density of filopodia, 0.60±0.09 per µm^2^ (N = 16). The lowest mean density of filopodia was recorded for cells that expressed GFP alone, 0.36±0.08 per µm^2^ (N = 9). Thus, fascin 2b and espin, separately or together, can modulate the lengths of filopodia and can also induce their formation.

### Phosphomimicry experiments indicate that the phosphorylation state of fascin 2b regulates formation of long filopodia

To determine if the phosphorylation state of fascin 2b at serine 38 influences the formation of long filopodia [21], two phosphomutant cDNAs were constructed that, when expressed, emulate the phosphorylated state because serine 38 in each was replaced with either a glutamate (GFP-S38E fascin 2b) or an aspartate (GFP-S38D fascin 2b) (**Figure 3R,S**). Phosphomutants such as these have a greatly reduced capacity to bind and bundle actin *in vitro* [21], [25]. In all transfected COS-7 cells expressing either of these phosphomutant proteins (number of cells examined for each protein, N = 200), no filopodia were observable (**Figure 3U,V,X,Y**). Because replacing serine 39 of fascin 1 with an alanine results in a constitutively active protein that binds and bundles actin [25], we anticipated similar behavior by phosphomutant GFP-S38A fascin 2b, in which serine 38 is replaced with an alanine. More specifically, this protein cannot be regulated by serine 38 phosphorylation and should retain its ability to bind and bundle actin [21]. 30% of cells (N = 211) that expressed GFP-S38A fascin 2b exhibited long filopodia (**Figure 3Q,T,W**). The percentage of cells that produced long filopodia when expressing GFP-S38A fascin 2b was smaller than the percentage of cells that developed long filopodia when GFP-WT fascin 2b was expressed, indicating that regulation of the serine's phosphorylation state may in fact influence the formation of filopodia.

### Fascin 2b and espin colocalize to filopodia *in vitro*, but colocalization is dependent on the phosphorylation state of fascin 2b

**Figure 4:**
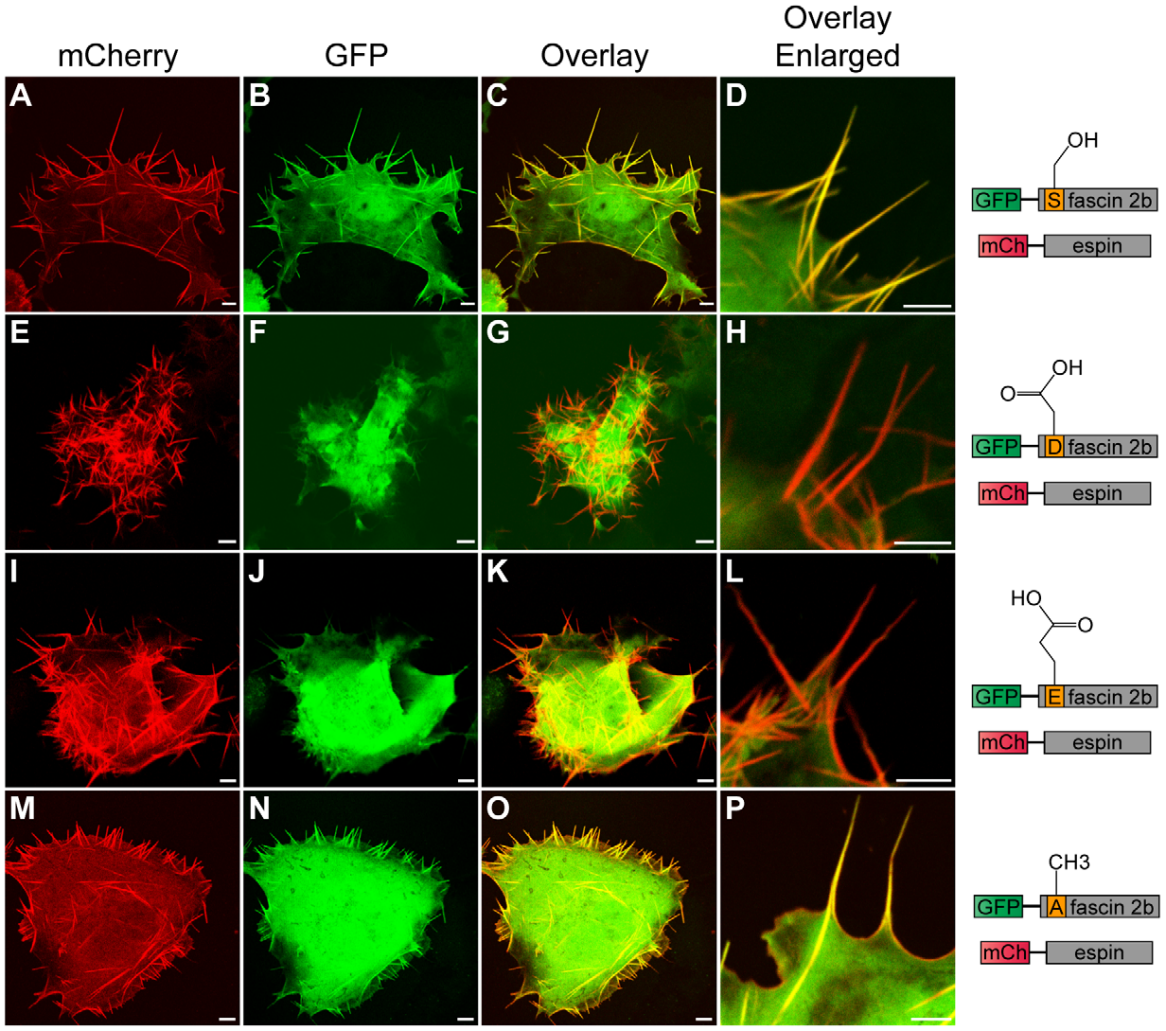
Subcellular localization patterns of wild-type or phosphomutant fascin 2b proteins in live COS-7 cells that coexpress espin. A cell (**A–D**) coexpressing GFP-WT fascin 2b and mCherry (mCh)-espin displays colocalization of these proteins in filopodia, which are detected as yellow in image overlays (**C,D**). Images of cells that coexpress mCherry-espin and either GFP-S38D fascin 2b (**E–H**) or GFP-S38E fascin 2b (**I–L**) reveal espin-laden protrusions (red) that lack the fascin 2b phosphomutant proteins (**G,H,K,L**). Micrographs of a cell that expresses both mCherry-espin and GFP-S38A fascin 2b (**M–P**) show that both proteins reside in filopodia (yellow) (**O,P**). Scale bars are 5 µm.

Because it is well established that espin is a major component of stereocilia [14] and we have shown that fascin 2b is present in these protrusions, we sought to determine the distribution of espin and fascin 2b in filopodia. COS-7 cells that coexpressed mCherry-espin and GFP-WT fascin 2b displayed evenly distributed colocalization of the fusion proteins in cellular protrusions; this codistribution frequently extended some distance below the surfaces of the cells (**Figure 4A–D**). COS-7 cells that coexpressed mCherry-espin with either phosphomutant protein, GFP-S38D fascin 2b (**Figure 4E–H**) or GFP-S38E fascin 2b (**Figure 4I–L**), displayed a robust formation of long filopodia laden with espin, but these protrusions lacked the phosphomutant proteins. This evidence indicates that phosphorylation of fascin 2b at serine 38 inhibits integration of this protein into espin-laced protrusions. In addition, phosphorylation of fascin 2b does not inhibit the formation of protrusions induced by espin. Finally, cells that simultaneously expressed GFP-S38A fascin 2b and mCherry-espin (**Figure 4M–P**) displayed results similar to cells that expressed GFP-WT fascin 2b and mCherry-espin (**Figure 4A–D**). These findings suggest that nonphosphorylated serine 38 is required for fascin 2b localization to espin-laden protrusions.

### Localization of fascin 2b to stereocilia is governed by phosphorylation

**Figure 5:**
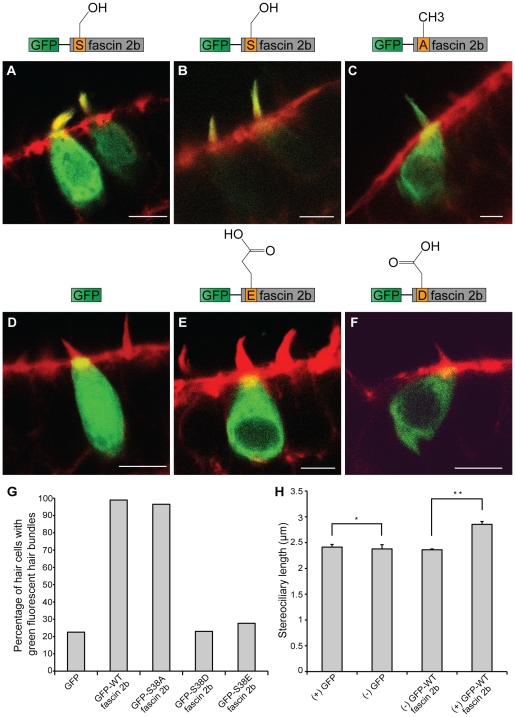
Localization patterns of wild-type or phosphomutant fascin 2b fusion proteins in hair cells, and the effect of GFP-WT fascin 2b expression on stereociliary length. Representative confocal images of transgenic hair cells in the anterior maculae of zebrafish at 4 dpf are displayed. Hair cells expressing GFP-WT fascin 2b (**A,B**), GFP-S38A fascin 2b (**C**), GFP (**D**), GFP-S38E fascin 2b (**E**), or GFP-S38D fascin 2b (**F**) are labeled with fluorophore-coupled phalloidin (red). The fusion proteins and GFP appear green. When high levels of GFP-WT fascin 2b are expressed in hair cells (number of cells, N = 192), the fusion protein localizes to the stereocilia and the somata (**A**), but when low levels are expressed, it localizes specifically to the hair bundles (**B**). GFP-S38A fascin 2b (N = 86) (**C**) localizes with a pattern that is similar to that of GFP-WT fascin 2b (**A**). In contrast, cells expressing GFP (N = 71) (**D**), GFP-S38E fascin 2b (N = 94) (**E**), or GFP-S38D fascin 2b (N = 74) (**F**) generally exhibit greatly reduced levels of GFP fluorescence in their hair bundles. Scale bars are 5 µm. Graph displays the percentage of transgenic hair cells with GFP fluorescence in their bundles for each expressed fluorescent protein (**G**). As represented by graph (**H**), cells that express GFP-WT fascin 2b ((+) GFP-WT fascin 2b, N = 132) have an increased mean hair-bundle length when compared to that of non-transgenic hair cells ((−) GFP WT-fascin 2b) in transgenic animals that mosaically express the transgene. The means of the bundle lengths of cells that lack expression of the fluorescent proteins ((−) GFP WT-fascin 2b, N = 480; (−) GFP, N = 45) and that of those that express GFP ((+) GFP, N = 148) are similar, indicating that there is no substantial experimental variation of the controls within and between the two groups. The means of the bundle lengths ± SEM are plotted. Single and double asterisks indicate *P* = 0.8653 and *P* = 0.0001, respectively, for Student's *t* tests.

Next, we sought to determine a role for serine 38 in targeting fascin 2b to stereocilia. To ascertain whether GFP-WT fascin 2b localizes to stereocilia (**Figure 5A,B**) in the anterior maculae of zebrafish larvae, we used somatic-cell transgenesis [43]. Hair cells that expressed high levels of the fusion protein, as determined by robust fluorescence using confocal laser-scanning microscopy, exhibited fascin 2b localization to stereocilia as well as somata. In contrast, cells that expressed lower levels of GFP-WT fascin 2b showed localization more specifically to the stereocilia. This may indicate that fascin 2b preferentially localizes to stereocilia when binding sites in the hair bundle are available and not saturated. Moreover, in greater than 98.9% (number of cells, N = 192) of cells that expressed GFP-WT fascin 2b, significant GFP fluorescence was observed in their hair bundles (**Figure 5G**). In contrast, when only GFP was introduced into hair cells as a control (**Figure 5D**), fewer than 22.5% (N = 71) of the cells had GFP fluorescence in their hair bundles, indicating that GFP-WT fascin 2b specifically localized to stereocilia. Similar to GFP-WT fascin 2b expression, when GFP-S38A fascin 2b was expressed (**Figure 5C**), 96.5% (N = 86) of cells had GFP fluorescence in their hair bundles (**Figure 5G**). This result was distinguished from those of transgenic hair cells that expressed GFP-S38E fascin 2b (**Figure 5E**) or GFP-S38D fascin 2b (**Figure 5F**). More specifically, for cells that expressed GFP-S38E fascin 2b or GFP-S38D fascin 2b, the percentage of cells that contained GFP in their bundles was either 27.7% (N = 94) or 23% (N = 74), respectively. These percentages were similar to those of cells that expressed GFP alone (**Figure 5G**). In conclusion, these results suggest that localization of fascin 2b to stereocilia is regulated by the phosphorylation state of serine 38.

### Overexpression of fascin 2b increases hair-bundle length

We next determined whether transgenic expression of GFP-WT fascin 2b in the hair cell influenced the length of the bundle. In these experiments, we measured the maximum length of each phalloidin-labeled hair bundle to determine the mean bundle length. The mean bundle length of cells that expressed GFP-WT fascin 2b (mean length ± SEM  = 2.85±0.06 µm; number of bundles, N = 132) under the control of a hair-cell promoter was compared to that of nonfluorescent hair cells in transgenic animals that were mosaic for transgene expression (2.36±0.02 µm, N = 480); a difference greater than 0.49 µm (*P* = 0.0001, Student's *t* test) was observed (**Figure 5H**). To show that this effect was attributable to fascin 2b and not to GFP, we also determined the mean bundle length of hair cells that expressed GFP alone (2.41±0.05 µm, N = 148). This mean was not significantly different from that of bundles from nontransgenic hair cells found in the wild-type cell population of mosaic transgenics (2.38±0.08 µm, N = 45, *P* = 0.8653) (**Figure 5H**). This study suggests a role for fascin 2b in establishing stereociliary length.

### Analyses of fascin 2a and 2b functions using morpholinos

In an effort to determine a possible role for fascin 2b in hair-bundle development, we used morpholino oligomers [44] to knockdown the production of this protein in zebrafish embryos. Morpholinos that target the *fascin 2b* mRNA start codon, the *fascin 2b* pre-mRNA exon 1-intron 1 splice donor site, or both were used in these studies. The maximum lengths of phalloidin-labeled hair bundles in the anterior maculae of injected embryos were determined using confocal microscopy. None of the morpholino injections significantly changed the mean bundle length when compared to that of animals injected with 5-bp mismatch control morpholinos (data not shown). To determine if fascin 2a played a role in establishing hair-bundle length, we used two different morpholinos that were directed towards *fascin 2a* pre-mRNA, and, again, we did not observe an effect. Finally, to determine if either of the fascin 2 isoforms were compensating for the other in animals in which a morpholino directed towards a single site was introduced, we attempted to knockdown fascin 2a and 2b expression simultaneously, and, yet again, hair-bundle length was not altered (data not shown). These results are not definitive because the morpholinos may not knockdown protein expression to levels low enough to produce an observable effect.

### Conclusions

Our data suggest that fascin 2b participates in bundling the actin filaments in stereocilia and, in so doing, influences hair-bundle morphology. We hypothesize that fascin 2b contributes in multiple ways to shape stereocilia. First, our data support the notion that fascin 2b and espin coordinate in stereocilia to lengthen these actin-based protrusions. This hypothesis is strengthened by our transfection experiments that used COS-7 cells; these studies demonstrated that coexpression of fascin 2b and espin substantially lengthened filopodia when compared to filopodia of cells that expressed each protein separately. In addition, we have shown that transgenic zebrafish hair cells overexpressing fascin 2b have significantly lengthened stereocilia when compared to wild-type cells. A potential factor involved in this lengthening may be that extensive actin cross-linking by fascin 2b decreases the rate of actin depolymerization that occurs towards the base of a stereocilium during actin treadmilling. Other relevant factors include the rate of actin polymerization that occurs towards the tip of a stereocilium and the constraint imposed on the protrusion's length by membrane tension [19].

Second, the presence of multiple cross-linking proteins may permit formation of the large actin bundles that are present in stereocilia. Recently, *in vitro* studies demonstrated that fascin 1 and espin together can organize actin into a thick bundle containing several hundred filaments, but fascin 1 alone can organize only up to 20 filaments of actin into a bundle [45]. If fascin 2b and fascin 1 behave similarly, this may suggest that fascin 2b may work with espin to form a thicker bundle of filamentous actin than either protein would be able to assemble individually.

Third, espin, fimbrin, and fascin 2b are regulated dissimilarly, implying different roles for these proteins in shaping stereocilia. Selection of different transcriptional start sites and alternative splicing of pre-mRNA result in the production of multiple espin isoforms [16], some of which localize with distinct patterns along the length of a stereocilium [17]. T-plastin, an isoform of fimbrin [12], [46], localizes to stereocilia in a temporally regulated manner during hair-cell development [12]. We showed by mutational analyses of fascin 2b that phosphorylation plays a significant role in the localization of this protein to stereocilia. Consequently, fascin 2b may assist in the formation and maintenance of the stereociliary taper. The characteristic shape of the taper is possibly the result of a balance between actin monomer addition towards the tip of a stereocilium and actin depolymerization in the region of the taper. The mechanism by which actin depolymerization occurs at the stereociliary taper during actin treadmilling is unknown. It is plausible that a kinase, residing in the taper region, phosphorylates local fascin 2b, which suppresses actin cross-linking, and, consequently, facilitates actin depolymerization. Identifying the relevant kinase and phosphatase and determining their possible locations in stereocilia will be central to understanding the role of fascin 2b in hair-bundle morphogenesis.

In summary, we identify fascin 2b as a highly specific member of stereocilia by immunolabeling studies. *In vitro* experiments demonstrate that fascin 2b organizes actin filaments into parallel bundles resembling those observed in stereocilia [8]–[10]. Our studies demonstrate that expression of fascin 2b in COS-7 cells results in the formation of long filopodia, and coexpression with espin produces even longer protrusions. Moreover, by the expression of fascin 2b phosphomutants in COS-7 cells, we show that formation of long filopodia and localization of fascin 2b to espin-laden protrusions are dependent on serine 38. Similarly, using transgenic zebrafish, we observe a reliance on residue 38 for consistent localization of fascin 2b to stereocilia. Finally, we note a significant lengthening of stereocilia as a result of overexpression of fascin 2b in hair cells. Together with observations that fascin 2 is a component of avian and murine stereocilia and that mice with a mutation in the cognate gene display hearing loss (Peter Gillespie, personal communication), our results indicate that fascin 2b and the orthologous proteins are important for stereociliary morphology across vertebrate species.

## Materials and Methods

### Zebrafish husbandry

Zebrafish of the Tübingen (Tü) strain were used in these studies. They were maintained at 28°C by standard procedures [47] and kept with the approval of the Case Western Reserve University Institutional Animal Care and Use Committee (Protocol Approval Number 2009-0167).

### Expression vectors

DNA manipulations were performed using standard procedures [48]. Enzymes for DNA manipulations were purchased from New England Biolabs, except where noted. All procedures involving kits were carried out according to the manufacturers' protocols.

Construction of the vector pMT/SV/PV/GFP-WT fascin 2b for the expression of GFP-WT fascin 2b in hair cells involved multiple steps. To create pMT/SV/PV, a multiple cloning site was generated by annealing [48] two oligonucleotides, 5′ MCS-pBSIISK+ and 3′ MCS-pBSIISK+. All oligonucleotides are listed in **Table 1**. The product was then ligated (T4 ligase; Promega) into pBluescript II SK(+) (Stratagene), which had been digested with *Spe*I and *Sac*II. The resulting construct was digested with *Not*I and *Afl*II to insert a polyadenylation addition sequence, which had been excised from pEGFP-1 (Clontech) using *Not*I and *Afl*II. The multiple cloning site with the polyadenylation addition sequence was removed with *Spe*I and *Bgl*II; this digested product was then ligated with the pminiTol2/MCS vector [49], which had been digested with the same enzymes, to create pMT/SV. The zebrafish *parvalbumin 3b* promoter, which drives expression in hair cells, was amplified from the Ppv3b-4 vector [43] by a PCR reaction (*Pfu* polymerase; Stratagene) that introduced *Bam*HI sites onto the product termini using primers Bam Pv3b 1 and 3′_no_G_Pv3b. The promoter was subcloned into pCRII-TOPO (Invitrogen), and the resulting plasmid was digested with *Bam*HI. The fragment containing the promoter was then ligated into *Bam*HI-digested pMT/SV, resulting in pMT/SV/PV. This was digested with *Age*I and *Xma*I, and a DNA fragment containing the GFP cDNA in frame with *fascin 2b* cDNA from pGFP:DrF2B [21], also digested with *Age*I and *Xma*I, was inserted to yield pMT/SV/PV/GFP-WT fascin 2b. To generate the GFP-S38D fascin 2b expression construct, the S38D fascin 2b cDNA was amplified by PCR from pGST:DrF2B S39D [21] using the primers F2B XhoI 5′ F2FD and F2B XmaI 3′ L2FD. The PCR product was digested with *Xho*I and *Xma*I and ligated into *Xho*I- and *Xma*I-digested pMT/SV/PV/GFP-WT fascin 2b vector.

**Table 1:** Oligonucleotides.

Primer Name	Primer Sequence (5′-3′)	Mutation Induced
a51g_g52a_c53g	AGGTGAACGCTTCAGCTCCAGAGCTCAAGAAGAAGCAGATCTG	S38E
a51g_g52a_c53g.1	CAGATCTGCTTCTTCTTGAGCTCTGGAGCTGAAGCGTTCACC	S38E
a51g_g52c.1	CTGCTTCTTCTTGAGGGCTGGAGCTGAAGCGTTCAC	S38A
a51g_g52a	GGTGAACGCTTCAGCTCCAGACCTCAAGAAGAAGCAGA	S38D
a51g_g52c	GTGAACGCTTCAGCTCCAGCCCTCAAGAAGAAGCAG	S38A
a51g_g52a.1	TCTGCTTCTTCTTGAGGTCTGGAGCTGAAGCGTTCACC	S38D
5′ MCS-pBSIISK+	CTAGTTTGGATCCTTAATTAAGTTTAAACAGGCGCGCCTGCGGCCGCACGCGTCTTAAGAGATCTCCGC	-
3′ MCS-pBSIISK+	GGAGATCTCTTAAGACGCGTGCGGCCGCAGGCGCGCCTGTTTAAACTTAATTAAGGATCCAAA	-
5′ Age mCherry	AAACCGGTACCATGGTGAGCAAGGGCGAGG	-
3′ RI mCherry	AAGAATTCCTTGTACAGCTCGTCCATGCCG	-
Bam Pv3b 1	AAGGATCCTTTGATTTCTTCATTTAAG	-
3′_no_G_Pv3b	TTGGATCCACCCGGGATATTCAAACTGTTGAGAGAATAAAACA	-
ZF 2B 5′ EX4	CGAGGACGAGCAGCTGATTCTGA	-
ZF 2B 3′ EX5.1	GTATTCCCAGAGGGAAGAGC	-
ZF fascin 2a 5′ start	ATGTCTACAAACGGAATAAGCGCA	-
ZF fascin 2a 3′ end	GTGCTCCCACAAGGATGAGGCA	-
ZF fascin 2b 5′ start	ATGCCCTCCAATGGCACCAAAGC	-
ZF fascin 2b 3′ end	TCAGTATTCCCAGAGGGAAGAGC	-
F2B XhoI 5′ F2FD	AACTCGAGCATGCCCTCCAATGGCACCAAAG	-
F2B XmaI 3′ L2FD	CTCCCGGGTCAGTATTCCCAGAGGGAAGAGC	-

To produce the plasmids pCRII::fascin 2a or pCRII::fascin 2b for the generation of each digoxigenin (DIG)-labeled RNA probe for *in situ* hybridization experiments, PCR amplification of *fascin 2a* cDNA or *fascin 2b* cDNA was conducted using the primer pairs ZF fascin 2a 5′ start and ZF fascin 2a 3′end or ZF fascin 2b 5′ start and ZF fascin 2b 3′end, respectively. The PCR products were separately subcloned into pCRII-TOPO. To construct pCMV::mCherry-espin, mCherry cDNA [50] was amplified by PCR from the template plasmid pRSET-B mCherry using the primers 5′ Age mCherry and 3′ RI mCherry, which added the restriction endonuclease sites for *Age*I and *Eco*RI to either end of the product. The product was then subcloned into pCR-BluntII-TOPO (Invitrogen) to create pCR::Age-mCherry-RI. The mCherry cDNA was then inserted into pCMV::GFP-espin, which contained small espin [34]. More specifically, the GFP and mCherry cDNAs were excised from their respective plasmids by digestion with *Age*I and *Eco*RI, and the mCherry cDNA was subsequently ligated into digested pCMV::GFP-espin from which GFP had been excised. The resulting vector was used to express mCherry-espin.

### Site-directed mutagenesis

The primers described in **Table 1** were used to generate *fascin 2b* cDNAs, which encode GFP-S38A fascin 2b, GFP-S38D fascin 2b, or GFP-S38E fascin 2b, for expression from modified versions of pCMV::GFP-fascin 2b [21]. Similarly, the primers listed in **Table 1** were used to generate *fascin 2b* cDNAs, which encode GFP-S38A fascin 2b or GFP-S38E fascin 2b, for expression from modified versions of pMT/SV/PV/GFP-WT fascin 2b. Appropriate substitutions were introduced by mutagenesis (QuikChange Lightning Site-Directed Mutagenesis Kit; Stratagene).

### *In situ* hybridization

Whole-mount *in situ* hybridizations were conducted on wild-type zebrafish larvae treated with 1-phenyl-2-thiourea to reduce pigmentation [26]. The plasmids pCRII::fascin 2a and pCRII::fascin 2b were used to generate antisense probes that recognize *fascin 2a* mRNA and *fascin 2b* mRNA, respectively. These plasmids were also used to synthesize labeled sense RNAs for control experiments. Frozen sections with a thickness of 16 µm were prepared from labeled embryos [51] that were immobilized in Optimum Cutting Temperature (OCT) Compound (Sakura Finetek).

### RT-PCR experiments

For RT-PCR experiments, cDNA was produced from adult zebrafish hair cells [26]. PCR amplifications were performed (*Ex Taq* DNA Polymerase; Takara Bio) with the interexonic primer pairs ZF 2B 5′ EX4 and ZF 2B 3′ EX5.1. The primers for these reactions recognized different exons of the *fascin 2b* gene and were used with PCR parameters designed to amplify a segment of the *fascin 2b* cDNA, but not the genomic locus.

### Fluorescent labeling of zebrafish

To label larvae with anti-fascin 2 serum and fluorophore-coupled phalloidin, 4-day-old larvae were fixed (Cytoskelfix; Cytoskeleton) for six minutes at −20°C and then processed according to standard procedures [21], [43]. The reagents used were anti-fascin 2 serum [21] diluted 1∶600, secondary antibody (Alexa Fluor 488 goat anti-rabbit IgG; Invitrogen) at a 1∶200 dilution, and an actin filament-labeling protein (Alexa Fluor 546 phalloidin; Invitrogen) at a 1∶50 dilution.

### Preparation of fascin 2b-filamentous actin complexes for electron microscopy

MBP-fascin 2b fusion protein was expressed in the *E. coli* strain BL21 and purified using amylose resin [21]. For actin-fascin 2b complex formation, rabbit skeletal muscle actin (Cytoskeleton) was incubated in G-buffer (5 mM Tris [pH 8.0], 0.2 mM CaCl_2_, 0.5 mM DTT, 0.2 mM ATP) on ice for 1 h at 2.5 mg/ml and then centrifuged at room temperature for 20 min at 14,000 × *g*; the supernatant was collected. The MBP-fascin 2b fusion protein stock was centrifuged at 100,000 × *g* for 1 h at 22°C; the supernatant was collected and its protein concentration determined. 5 µM MBP-fascin 2b was combined with 2.4 µM G-actin in G-buffer. Actin polymerization was stimulated by adding filamentous buffer (500 mM KCl, 20 mM MgCl_2_, 10 mM ATP), at a volume 1/10^th^ of the final reaction volume, to the G-buffer that contained the proteins.

### Negative staining and transmission electron microscopy

5 µl of MBP-fascin 2b-filamentous actin complexes were placed on a 400-mesh glow discharge/carbon-coated copper grid. After 1 min, the grid was washed with water and then stained with 1% uranyl acetate in water. After 2 min, excess fluid was removed from the grid. Samples were viewed with a transmission electron microscope (FEI Tecnai F30; FEI).

### Supernatant-depletion assay

The fascin 2b-filamentous actin interaction was characterized using the supernatant-depletion assay [38]. Purified MBP-fascin 2b was dialyzed against G-buffer and then centrifuged at 100,000 × *g* for 1 h to remove precipitated protein. Lyophilized rabbit skeletal muscle actin was resuspended in G-buffer overnight at 4°C and then centrifuged at 14,000 × *g* for 20 min to allow depolymerization and the subsequent removal of aggregated protein. In all experiments, 0.1 µM fascin 2b fusion protein was used. In contrast, the actin concentration was varied from 0–20 µM between each experiment. The two different proteins were combined, and then actin polymerization was initiated at room temperature by the addition of filamentous buffer using an amount 1/10^th^ of the total reaction volume. After 1 h, free actin monomers and free MBP-fascin 2b were separated from free filamentous actin and MBP-fascin 2b-filamentous actin complexes by centrifugation at 100,000 × *g* for 40 min at 22°C. Samples of supernatants and pellets were separated by SDS-PAGE. Gels that contained either resuspended pellets or supernatants were stained (SYPRO Ruby Protein Gel Stain; Invitrogen). Proteins from additional gels containing the supernatants were transferred to nitrocellulose membranes (Odyssey Nitrocellulose Membrane; LI-COR Biosciences). The membranes were analyzed in western blot analyses using purified anti-fascin 2 serum [21], diluted 1∶600, as the primary antibody and donkey anti-rabbit serum linked to Cy-5, diluted 1∶5000, as the secondary antibody (Millipore). Fluorescence intensities of the protein bands in the gels or on the membranes were determined using a multimode scanner (Typhoon 9400; GE Healthcare) and analyzed with software (ImageQuantTL; GE Healthcare). Because we were working with lyophilized actin monomers that we polymerized at different concentrations, it was necessary to determine the percentage of actin that polymerized at each concentration and to use these values to calculate the *K*_d_. More specifically, the band intensities of actin from the supernatants and pellets, which represented the monomeric and filamentous actin populations, respectively, were compared. The fraction of actin that polymerized at each concentration was determined. Each appropriate fraction was multiplied by the initial concentration of actin in each solution to determine the concentration of filamentous actin present. Bound MBP-fascin 2b was calculated from the amount depleted from the supernatants. Results were plotted as bound fascin 2b versus free filamentous actin and were fit according to Bryce et al. [38]. Graphing and statistical analyses were performed with Prism software (GraphPad Software).

### Tissue culture and cell transfection

COS-7 cells (American Type Culture Collection) were cultured at 37°C in Dulbecco's Modified Eagle Medium (D-MEM; Invitrogen) supplemented with 100 units/ml penicillin, 100 µg/ml streptomycin, and 10% fetal bovine serum. Cells were cultured on glass coverslips or on glass-bottom dishes (MatTek) for 2–3 days and then transfected. The transfection mixture, containing 4 µg of DNA and 4 µl of transfection reagent (Lipofectamine 2000; Invitrogen) in 500 µl of media (Opti-MEM I Reduced Serum Media; Invitrogen), was incubated with the cells. Live cells were imaged 24–35 h after transfection. Five filopodia, randomly selected from each cell, were measured for length. Images of COS-7 cells were acquired on a confocal laser-scanning microscope (Leica) equipped with a 100× objective lens. In experiments involving espin expression, cells were transfected with either pCMV::mCherry-espin, pEGFP-espin [34], or pcDNA3-espin [52].

### Scanning electron microscopy

Cell monolayers were fixed overnight at room temperature using a solution containing 4% paraformaldehyde (Electron Microscopy Sciences) and 2.5% glutaraldehyde (Electron Microscopy Sciences) in phosphate-buffered saline (PBS). Samples were then washed with PBS at room temperature. Next, the samples were exposed to 1% osmium tetroxide for 30–60 min at room temperature. The samples were washed with distilled water six times, and then they were dehydrated through a graded series of ethanol solutions: 30%, 50%, 70%, 95%, and 100% ethanol. They were next washed in a 1∶1 solution of ethanol:hexamethyldisilazane (HMDS) and then in 100% HMDS. Cell images were collected using a scanning electron microscope (JSM 5310; JEOL Ltd).

### Generation, labeling, and imaging of transgenics

To generate somatic cells expressing transgenes in zebrafish, we used the protocol described by Balciunas et al.; however, we injected the zebrafish embryos with 100 pg of each DNA construct [49]. For labeling the hair bundles of transgenic animals at 4 dpf, larvae were fixed with 4% paraformaldehyde in PBS overnight at 4°C and conventional techniques were used with actin-filament labeling reagent (Alexa Fluor 546 phalloidin) [43]. A method similar to Peng et al. was used to measure the lengths of phalloidin-labeled hair bundles [40]. Briefly, to measure the maximum lengths of hair bundles, all images were acquired using a confocal laser-scanning microscope (Leica) equipped with a 40× objective lens and visualized using the manufacturer's software. For each anterior macula studied, a stack of images captured in the *z*-plane was collected and compiled into image sequences using Volocity software (Improvision). The maximum length of each phalloidin-labeled hair bundle was measured in the *xy*-plane. Statistical analyses were performed using Microsoft Office Excel (Microsoft).

### Supplemental materials and methods

The procedures used to produce the supplemental data are in **Text S1**.

## Supporting Information

Figure S1 Evaluation of fascin 2 mRNAs and hepatocyte mRNAs in adult zebrafish hair cells by RT-PCR analyses. An agarose gel reveals that liver transcripts of *apolipoprotein A-I* (lane 1) and *apolipoprotein Eb* (lane 2 and 3) are undetectable in hair cells. These amplifications were attempted with primer pairs apoA-I F2 and apoA-I R2 (lane 1), apoEb F1 and apoEb R1 (lane 2), or apoEb F2 and apoEb R2 (lane 3). In contrast, *fascin 2a* mRNA (lane 4) and *fascin 2b* mRNA (lane 5) are both shown to be present in adult hair cells. These amplifications were conducted using primer pairs zf F2A 5′EX4.1 and zf F2A 3′EX5 (lane 4) or zf F2B 5′E3.1 and zf F2B 3′E4.1 (lane 5).
(1.42 MB TIF)

Figure S2 Detection of the levels of fascin 2 transcripts in hair cells using absolute quantitative real-time PCR. Graph shows levels of transcripts for *fascin 2a*, *fascin 2b*, and *beta-2 microglobulin* as measured using absolute quantitative real-time PCR (A). Each column represents a threshold cycle (Ct) value measured using primers directed towards *fascin 2a* cDNA, *fascin 2b* cDNA, or *beta-2 microglobulin* cDNA, with adult hair-cell cDNA used as a template. The average Ct value for *fascin 2a* cDNA and *fascin 2b* cDNA is 30.99±0.11 (mean ± SEM) and 27.30±0.15, respectively (B). The number of copies of each fascin 2 cDNA per μl of total hair-cell cDNA is shown (C). mRNA levels in the hair cell are proportional to calculated cDNA copy numbers. The concentration of *fascin 2b* cDNA (1612 copies per μl of hair-cell cDNA) is approximately 44 times greater than that of *fascin 2a* cDNA (37 copies per μl of hair-cell cDNA).
(4.26 MB TIF)

Figure S3 Localization of fascin 2b and espin proteins in fixed COS-7 cells. Schematics of the proteins expressed in cells are portrayed: GFP-WT fascin 2b (A), GFP (B), GFP-espin (C), and GFP-WT fascin 2b and mCherry-espin (G). To visualize actin-based filopodia, cells were labeled with Alexa 568 phalloidin (red)(D-F) or Alexa 633 phalloidin (blue) (J,K). Merged images show localization of GFP-WT fascin 2b (green) (D) or GFP-espin (green) (F) to filopodia where they overlap with actin (yellow). No filopodia are detectable in a fixed cell that expresses only GFP (green) (E). Coexpression of GFP-WT fascin 2b (H) and mCherry-espin (I) shows that both proteins colocalize (white) (K) to phalloidin-labeled filopodia (J) in a fixed COS-7 cell. Scale bars are 5 μm.
(3.71 MB TIF)

Text S1 Supplemental Materials and Methods.
(0.10 MB PDF)
