# Supplementary figures and images for: Fascin 2b Is a Component of Stereocilia that Lengthens Actin-Based Protrusions
Source: PLoS One. 2011 Apr 26;6(4):e14807. doi: 10.1371/journal.pone.0014807 (PMC3082522; doi:10.1371/journal.pone.0014807)

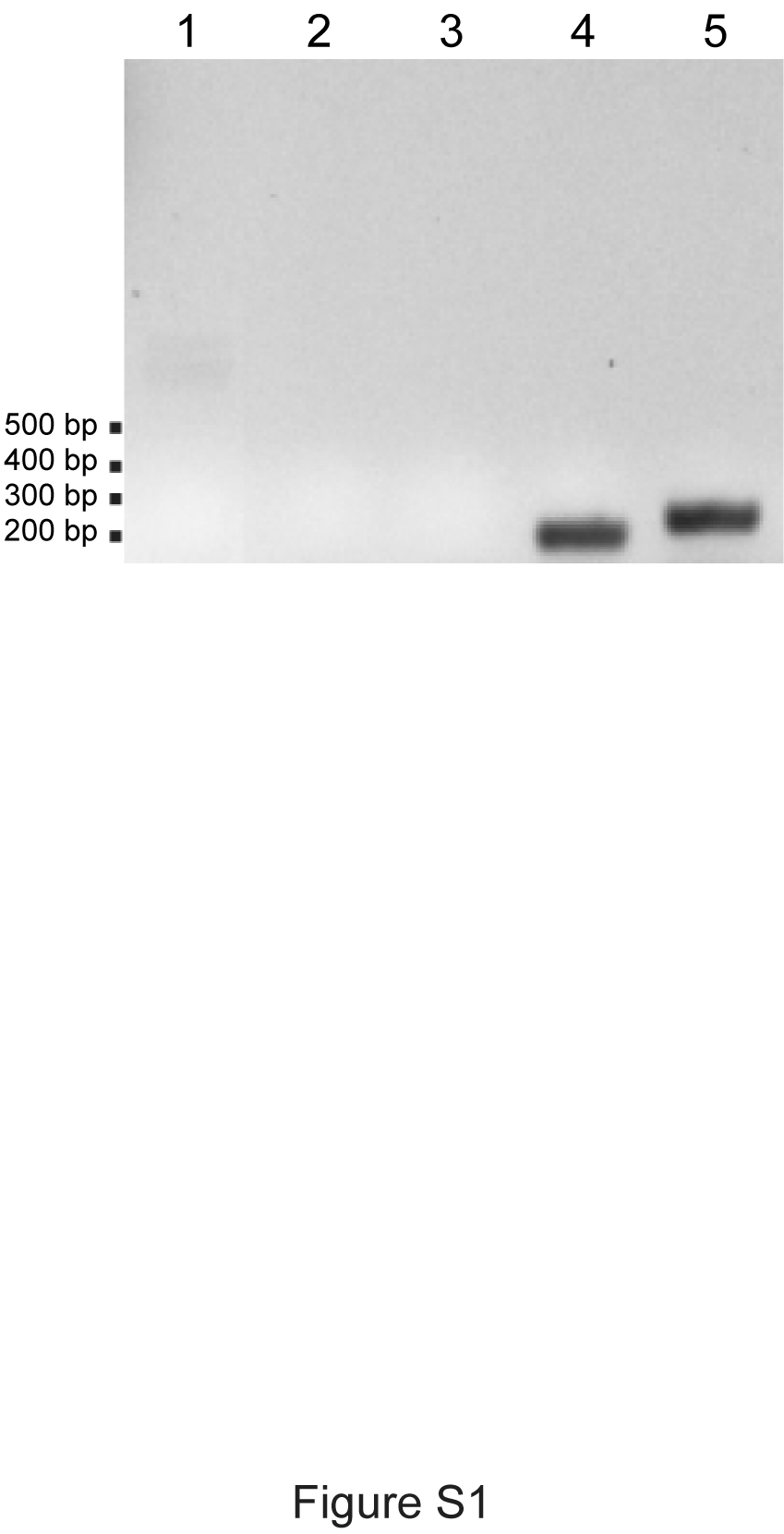

Supplement: Figure S1 — Evaluation of fascin 2 mRNAs and hepatocyte mRNAs in adult zebrafish hair cells by RT-PCR analyses. An agarose gel reveals that liver transcripts of apolipoprotein A-I (lane 1) and apolipoprotein Eb (lane 2 and 3) are undetectable in hair cells. These amplifications were attempted with primer pairs apoA-I F2 and apoA-I R2 (lane 1), apoEb F1 and apoEb R1 (lane 2), or apoEb F2 and apoEb R2 (lane 3). In contrast, fascin 2a mRNA (lane 4) and fascin 2b mRNA (lane 5) are both shown to be present in adult hair cells. These amplifications were conducted using primer pairs zf F2A 5′EX4.1 and zf F2A 3′EX5 (lane 4) or zf F2B 5′E3.1 and zf F2B 3′E4.1 (lane 5). (1.42 MB TIF) [file pone.0014807.s001.tif]

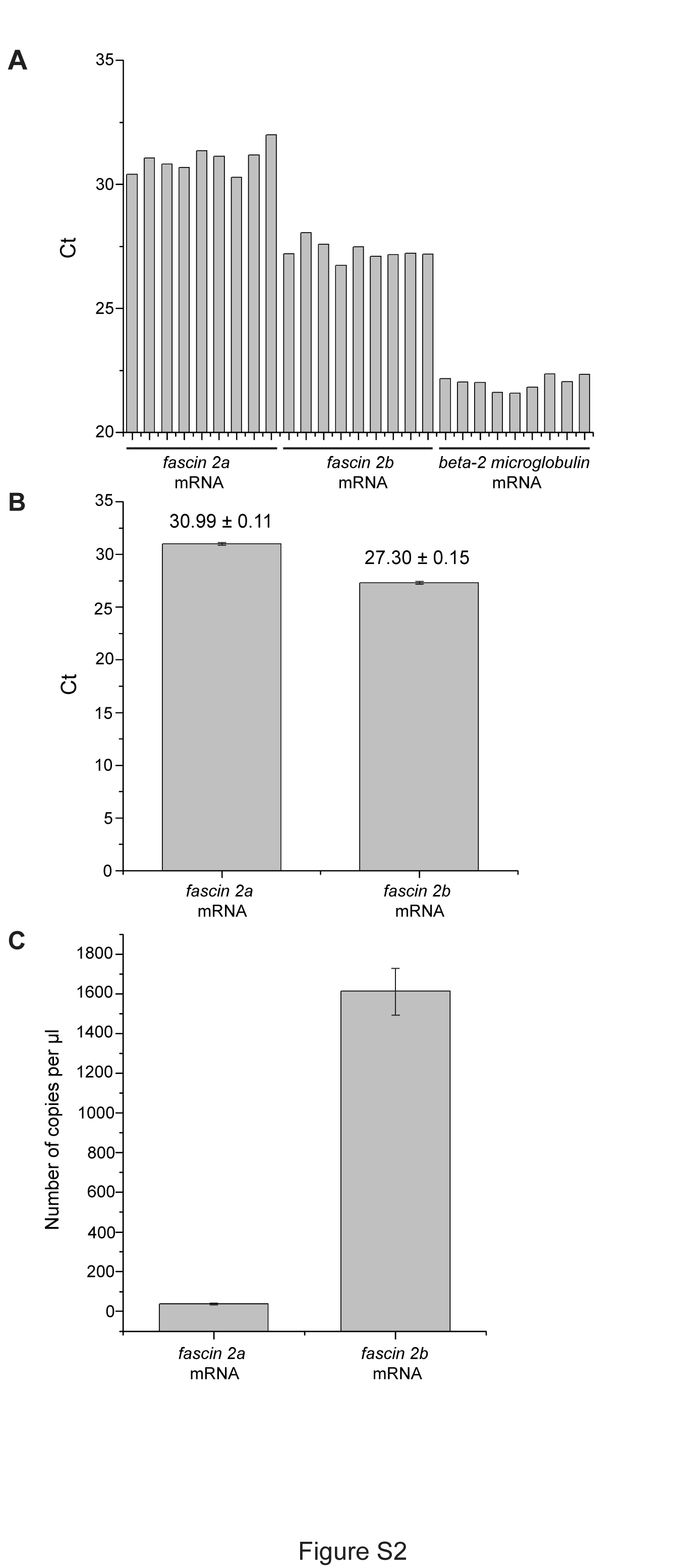

Supplement: Figure S2 — Detection of the levels of fascin 2 transcripts in hair cells using absolute quantitative real-time PCR. Graph shows levels of transcripts for fascin 2a, fascin 2b, and beta-2 microglobulin as measured using absolute quantitative real-time PCR (A). Each column represents a threshold cycle (Ct) value measured using primers directed towards fascin 2a cDNA, fascin 2b cDNA, or beta-2 microglobulin cDNA, with adult hair-cell cDNA used as a template. The average Ct value for fascin 2a cDNA and fascin 2b cDNA is 30.99±0.11 (mean ± SEM) and 27.30±0.15, respectively (B). The number of copies of each fascin 2 cDNA per μl of total hair-cell cDNA is shown (C). mRNA levels in the hair cell are proportional to calculated cDNA copy numbers. The concentration of fascin 2b cDNA (1612 copies per μl of hair-cell cDNA) is approximately 44 times greater than that of fascin 2a cDNA (37 copies per μl of hair-cell cDNA). (4.26 MB TIF) [file pone.0014807.s002.tif]

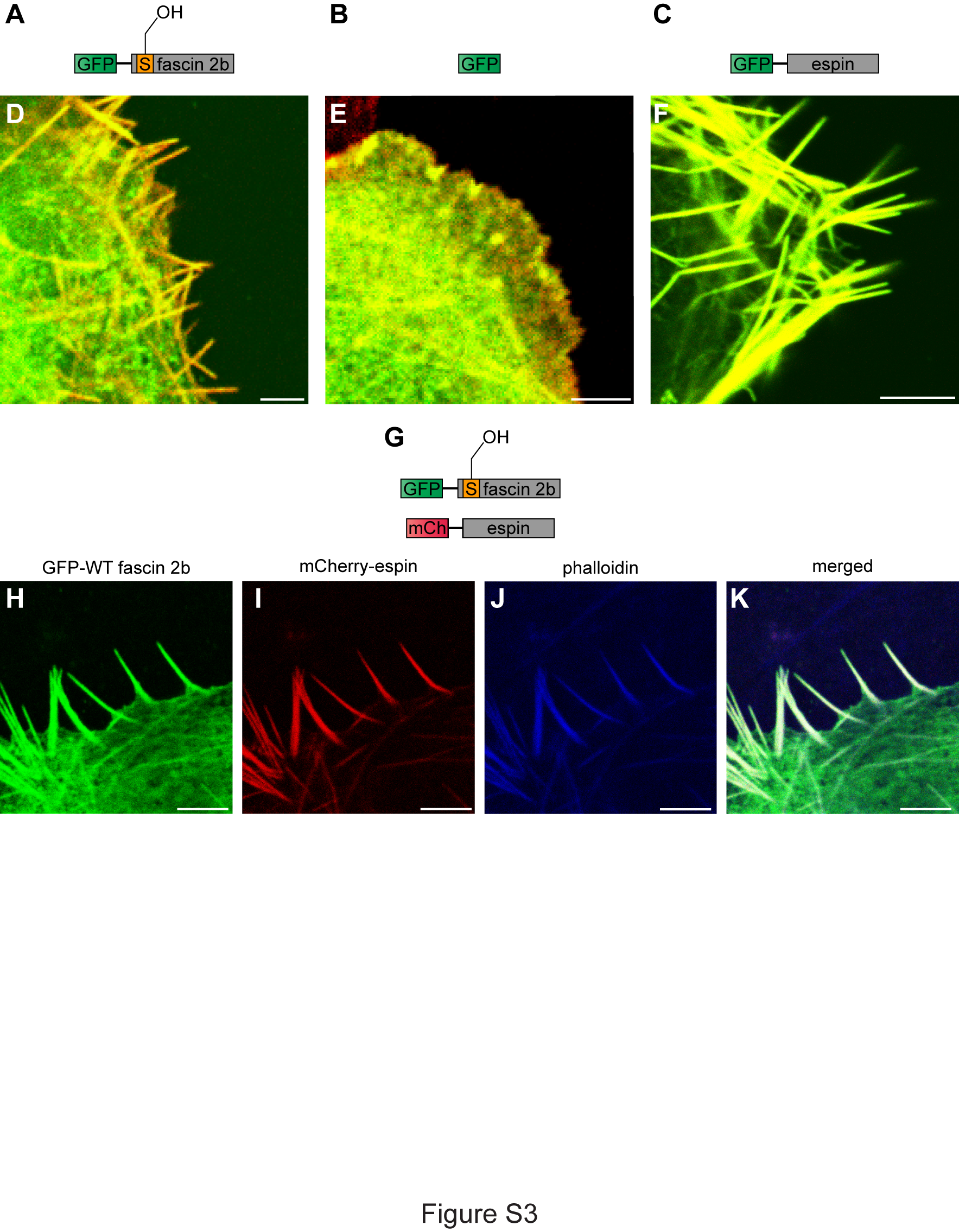

Supplement: Figure S3 — Localization of fascin 2b and espin proteins in fixed COS-7 cells. Schematics of the proteins expressed in cells are portrayed: GFP-WT fascin 2b (A), GFP (B), GFP-espin (C), and GFP-WT fascin 2b and mCherry-espin (G). To visualize actin-based filopodia, cells were labeled with Alexa 568 phalloidin (red)(D-F) or Alexa 633 phalloidin (blue) (J,K). Merged images show localization of GFP-WT fascin 2b (green) (D) or GFP-espin (green) (F) to filopodia where they overlap with actin (yellow). No filopodia are detectable in a fixed cell that expresses only GFP (green) (E). Coexpression of GFP-WT fascin 2b (H) and mCherry-espin (I) shows that both proteins colocalize (white) (K) to phalloidin-labeled filopodia (J) in a fixed COS-7 cell. Scale bars are 5 μm. (3.71 MB TIF) [file pone.0014807.s003.tif]
