# Supplementary material for: Fascin 2b Is a Component of Stereocilia that Lengthens Actin-Based Protrusions
Source: PLoS One. 2011 Apr 26;6(4):e14807. doi: 10.1371/journal.pone.0014807 (PMC3082522; doi:10.1371/journal.pone.0014807)
Supplement: Text S1 — Supplemental Materials and Methods. (0.10 MB PDF) [file pone.0014807.s004.pdf]

Supplemental Materials and Methods

Absolute quantitative real-time PCR. Primers were designed to recognize *fascin 2a* cDNA (zf F2A 5'EX4.1 and zf F2A 3'EX5), *fascin 2b* cDNA (zf F2B 5'E3.1 and zf F2B 3'E4.1), and *beta-2 microglobulin* cDNA (zf b2m qPCR F and zf b2m qPCR R), a product of a housekeeping gene. The primers were used in absolute quantitative real-time PCR studies and to produce plasmids to generate standard curves for these experiments [1]. To generate the plasmids, PCR amplifications were performed using hair-cell cDNA [2] as template. The product of each reaction was subcloned separately into the pCR8/GW/TOPO vector (Invitrogen). The identities of the PCR products were confirmed by DNA sequencing. Standard curves were generated, using defined quantities of these plasmids, to determine the number of copies of each cDNA in question in the pool of hair-cell cDNAs [2]. The concentrations of the purified DNA plasmids were measured by spectrophotometry (NanoDrop; Thermo Scientific). Plasmids containing *fascin 2a* cDNA, *fascin 2b* cDNA, or *beta-2 microglobulin* cDNA were serially diluted to obtain three standard series ranging from 1×10^7 copies per µl to 1×10^2 copies per µl [1].

PCR amplifications conducted in absolute quantitative real-time PCR experiments were performed in triplicate using hair-cell cDNA [2] or the plasmid standards (SYBR Green PCR Master Mix; Applied Biosystems). Each reaction contained 12.5 µl of SYBR Green PCR Master Mix, 1 µl each of forward and reverse primer (20 µM), 2 µl of hair-cell cDNA, and 8.5 µl of nuclease-free water. For non-template controls, nuclease-free water was substituted for cDNA. The reactions were performed (7300 Real-Time PCR System; Applied Biosystems) with the following PCR parameters: 10 min at 95°C followed by 40 cycles of 15 s at 95°C, 15 s at 55°C, and 40 s at 72°C. Fluorescence

signals were collected during the elongation phase. A dissociation cycle was used as the final cycle of the reaction: 15 s at 95°C, 1 min at 60°C, 15 s at 95°C, and 15 s at 60°C.

Three standard curves were each plotted using the natural logs of the number of copies per μl on the abscissa and the threshold cycle (Ct) values on the ordinate (7300 Real-Time PCR System; Applied Biosystems). With the linear trendlines determined as $y = -3.1777x + 35.909$, $y = -3.5936x + 38.787$, and $y = -3.5684x + 39.012$, the primer efficiencies were calculated to be 106%, 89.7%, and 90.7% for *fascin 2a*, *fascin 2b*, and *beta-2 microglobulin* cDNA amplicons, respectively. The dissociation curves of the non-template controls showed flat profiles (data not shown). The remaining aspects of the absolute quantitative real-time PCR protocol were carried out according to Leong and colleagues [1].

Morpholino experiments. Morpholinos (Genetools) intended to block *fascin 2b* mRNA translation (f2b-AUG: 5'-CTTTGCTGCCATTGGAGGGCATCCT-3') or *fascin 2b* pre-mRNA splicing (f2b-e1i1: 5'-GTACACCAGCACAACTTACCCTGTC-3') were designed to anneal to the start codon or the exon 1-intron 1 junction, respectively. 5-bp mismatch morpholinos intended not to anneal to *fascin 2b* mRNA (f2b-AUG-mis#2: 5'-CTTTCCTCCCATTCGAGGCCATGCT-3') or *fascin 2b* pre-mRNA (f2b-e1i1-mis: 5'-GTAGAGCAGGACAACTTACCGTCTC-3') targets were used in these experiments as controls. Two morpholinos intended to block splicing of *fascin 2a* pre-mRNA were designed to target either the exon 1-intron 1 junction (f2a-e1i1: 5'-ACACACAATCACCGCTTACCTTGAC-3') or the exon 2-intron 2 junction (f2a-e2i2: 5'-TCAACCAACATGCTCTTACATTTCC-3'). One- or two-cell stage zebrafish

embryos were injected with 1 nl of cocktail containing 0.5 mM morpholino prepared with Danieau's solution and phenol red [3]. Additionally, as controls in each experiment, animals were analyzed that each had been injected with a cocktail containing Danieau's solution and phenol red.

Labeling zebrafish. In morpholino experiments, fish, at 3 dpf, that had been injected were fixed with 4% paraformaldehyde in PBS. Next, they were permeabilized overnight at room temperature with 3% Triton X-100 in PBS. Subsequently, they were blocked with 5% goat serum in PBS for 2-6 h at room temperature and then incubated overnight at 4°C with a 1:800 dilution of primary antibody directed against parvalbumin 3 [4], an abundant calcium-binding protein in hair cells. The fish were rinsed with 5% goat serum four times over a period of 5 to 6 h and then incubated overnight at 4°C with secondary antibody (Alexa Fluor 546 goat anti-rabbit IgG; Invitrogen) diluted 1:200 in 5% goat serum. Next, the fish were rinsed with 5% goat serum in PBS, once for 5 min and then again for 30 min. Hereafter, labeled phalloidin (Alexa Fluor 488 phalloidin; Invitrogen), diluted 1:60 in 5% goat serum, was incubated with the fish overnight at 4°C. Following two washes, each for 1 h, with 5% goat serum, labeled fish were imaged while in fluorescence mounting medium (Vectashield; Vectorlabs). The bundle lengths of hair cells in the anterior maculae of the control animals were compared with those that were injected with the experimental morpholinos. The maximum length of each phalloidin-labeled hair bundle was measured using the protocol described in the materials and methods section.

Fixed-cell imaging. Cells were fixed 24 h after transfection in 4% paraformaldehyde, permeabilized using 0.1% Triton X-100 in PBS, blocked with 1% bovine serum albumin (BSA) in PBS, counterstained with phalloidin (Alexa Fluor 546 phalloidin or Alexa Fluor 633 phalloidin; Invitrogen) in 1% BSA diluted in PBS, and mounted in fluorescence mounting media (Vectashield; Vectorlabs). Images of COS-7 cells were acquired on a confocal laser-scanning microscope (Leica) equipped with a 100× objective lens.

The primer pairs are listed:

zf F2A 5'EX4.1: 5'-TCGTCCCATCCTGGTCCTGCG-3'

zf F2A 3'EX5: 5'-AGACCACTGCTGGAGACGTACC-3'

zf F2B 5'E3.1: 5'-TGGCACTGCGGGCCAGCAATG-3'

zf F2B 3'E4.1: 5'-CATAAACCGAGCGACTGGCGTC-3'

zf b2m qPCR F: 5'-GCCTTCACCCCAGAGAAAGG-3'

zf b2m qPCR R: 5'-GCGGTTGGGATTTACATGTTG-3'

apoA-I F2: 5'-ATCCAAGCTGGAGCCCCACCGTG-3'

apoA-I R2: 5'-ATGCCTGGATGGCCTTGGCGATG-3'

apoEb F1: 5'-GGCTCGTAGCCTGTTCCAGGCT-3'

apoEb R1: 5'-GTAGGTTGCTACGGTGTTGCGG-3'

apoEb F2: 5'-TGCCTCTGATGCTGCTGGTCAG-3'

apoEb R2: 5'-AGAGGTGCGTAGGTTCTCGGC-3'

References

1. Leong DT, Gupta A, Bai HF, Wan G, Yoong LF, et al. (2007) Absolute quantification of gene expression in biomaterials research using real-time PCR. Biomaterials 28: 203-210.

2. McDermott BM, Jr., Baucom JM, Hudspeth AJ (2007) Analysis and functional evaluation of the hair-cell transcriptome. Proc Natl Acad Sci U S A 104: 11820-11825.

3. Yuan S, Sun Z (2009) Microinjection of mRNA and morpholino antisense oligonucleotides in zebrafish embryos. J Vis Exp 27: 1113.

4. Heller S, Bell AM, Denis CS, Choe Y, Hudspeth AJ (2002) Parvalbumin 3 is an abundant Ca^{2+} buffer in hair cells. J Assoc Res Otolaryngol 3: 488-498.
